# Dynamic patterns of healthy lifestyle awareness after COVID-19: a study using Google Trends and joinpoint regression

**DOI:** 10.3389/fdata.2025.1717592

**Published:** 2026-01-13

**Authors:** Zahroh Shaluhiyah, Shabrina Arifia Qatrannada, Roshan Kumar Mahato, Farid Agushybana, Sri Handayani, Dzul Fahmi Afriyanto, Usha Rani, Dewie Sulistyorini

**Affiliations:** 1 Department of Health Promotion, Faculty of Public Health, Diponegoro University, Semarang, Indonesia; 2 Department of Health Management Innovative Technology, Faculty of Public Health, Khon Kaen University, Khon Kaen, Thailand; 3 Department of Biostatistics and Demography, Faculty of Public Health, Diponegoro University, Semarang, Indonesia; 4 Department of Epidemiology, Faculty of Public Health, Dian Nuswantoro University, Semarang, Indonesia; 5 Department of Health Care and Hospital Management, Prasanna School of Public Health, Manipal, India; 6 Institute of Biomedical and Health Sciences, Hiroshima University, Higashihiroshima, Japan

**Keywords:** digital health surveillance, Google Trends, information seeking behavior, joinpoint regression, public health awareness

## Abstract

**Introduction:**

The COVID-19 pandemic has significantly influenced public interest in health-related behaviors, as reflected in online search trends. Analyzing these trends provides insights into shifting health concerns and informing future public health strategies. This study examined Google Trends data to assess the changes in public interest in mental health, healthy diet, sleep, screen time, physical activity, and tobacco smoking before, during, and after the COVID-19 pandemic.

**Methods:**

Google Trends data (2019–2023) were analyzed using joinpoint regression to identify statistically significant shifts in relative search volume (RSV) over time. Additionally, the Mann–Whitney U test was conducted to examine differences in mean RSV across time period.

**Results:**

Awareness that consistently increased during and after the pandemic was observed in mental health, particularly anxiety, and sleep patterns. These topics showed significant positive trends in joinpoint regression and higher mean RSVs, with statistically significant differences across time periods (*p* < 0.05). In contrast, some behaviors such as physical activity and screen time saw increased awareness only during the pandemic but did not sustain afterward. Whilst, dietary behavior and smoking either remained stagnant or declined, indicating limited or declining public interest despite their relevance to health outcomes.

**Conclusion:**

Digital interest in health behaviors varied during and after COVID-19, with only mental health and sleep showing sustained concern. However, spikes in awareness often reflected personally relevant issues, highlighting Google Trends' potential as an early signal for health promotion efforts.

## 1 Introduction

A significant portion of the developing world is undergoing an epidemiological transition characterized by a triple disease burden. This encompasses an increasing prevalence of non-communicable diseases (NCDs), continued presence of infectious diseases, growing impact of accidental injuries, and globalization (NCD Child, 2019). In Indonesia, social, demographic, and economic changes have occurred unevenly, resulting in the coexistence of various stages of this transition across regions (Ministry of Finance Indonesia, 2020). These shifts pose significant challenges to achieving Sustainable Development Goals that aim to ensure healthy lives and promote well-being for all ages (UNICEF, 2020).

The COVID-19 pandemic has had an unprecedented impact on nearly every aspect of life worldwide by reshaping daily routines, priorities, and health concerns (World Health Organization, 2020). Beyond the immediate threat posed by the virus, the pandemic has triggered significant lifestyle changes with far-reaching implications for physical, mental, and social (de Palma et al., 2022; Manchia et al., 2022). These changes underscore the importance of addressing key determinants of health behavior, including physical activity, mental health, healthy diet, substance use, sedentary behavior (e.g., screen time), and sleep patterns collectively referred to as “the big six” (Gardner et al., 2022, 2023). These behaviors play pivotal roles in determining short- and long-term health outcomes, and are recognized as major contributors to the global burden of NCDs (Akseer et al., 2020; Budreviciute et al., 2020).

As individuals adapt to prolonged lockdowns, social distancing, and heightened health concerns, many have turned to online platforms for information on maintaining healthy lifestyles in the face of new challenges (Nawaz et al., 2024). In this context, understanding shifts in online health information-seeking behavior has become essential for public health researchers and practitioners striving to promote healthy living in the post-pandemic world. Prior work on diffusion processes shows that even when underlying behavioral or social contact networks cannot be directly observed, population-level “states” captured through digital traces can still provide meaningful insights into how concerns and behaviors propagate (Sefer and Kingsford, 2015, 2016). The surge in digital health information-seeking has highlighted the role of online search engines, particularly Google, as a primary resource for health-related knowledge (Bachl et al., 2024; Jacobs et al., 2017). Google Trends, a tool that tracks the frequency and distribution of search queries over time, provides a unique window for public interest and engagement with health topics (Nuti et al., 2014). By analyzing Google Trends data, researchers can observe trends in public behavior and identify patterns in information-seeking related to these critical health determinants.

In Indonesia, where traditional surveillance systems for health behaviors are often limited, delayed, or costly to implement at scale, digital trace data offer an accessible and timely proxy to understand population-level awareness. This is particularly relevant in the context of NCD prevention, where awareness and early recognition of healthy lifestyle practices represent the earliest stages of behavior change. Population search interest has been widely used as a first signal of shifting awareness, allowing researchers to detect emerging concerns long before they translate into measurable clinical or behavioral outcomes.

During the COVID-19 pandemic, such insights have been invaluable for understanding population concerns, motivations, and needs, especially in areas where conventional health information delivery channels are limited (McClaughlin et al., 2023; Porat et al., 2020). Similar to how SEIR-based diffusion models infer underlying spread patterns despite incomplete data, digital search activity can serve as an accessible early indicator of emerging public concerns (Sefer, 2022). Despite this potential, there remains a notable gap in Indonesia regarding longitudinal, time-series evidence on how public awareness of healthy lifestyle determinants evolved during and after the pandemic. Most available studies are cross-sectional or focus narrowly on clinical outcomes, leaving the temporal dynamics of awareness insufficiently explored.

This study explored the influence of the COVID-19 pandemic on healthy lifestyle information-seeking behaviors by leveraging Google Trends data. Specifically, it examines how lockdowns, vaccination rollouts, and other pandemic milestones correlate with spikes in health-related searches, thereby shedding light on evolving health priorities during global crises (Filip et al., 2022; Pollard et al., 2020). By aligning these insights with “the big six” determinants of health, this analysis contributes to a broader understanding of how public health priorities shifted during the pandemic. Furthermore, it underscores the importance of evidence-based interventions to address lifestyle factors exacerbated by the pandemic (Chavira et al., 2022; Su and Jin, 2023). This study offers valuable insights for public health policymakers, educators, and digital health professionals, helping identify emerging areas of public concern, tailor health communication strategies, and support the development of targeted interventions.

By focusing on popular search terms associated with healthy lifestyles, this study aimed to assess how public interest in these topics fluctuated during the key phases of the pandemic. This work conceptualizes online information-seeking as a proxy for population-level awareness, rather than as a predictor of clinical behavior, reflecting the understanding that awareness often precedes action and that digital search activity can signal emerging shifts in public concern. The study offers insights into how infodemiologic data can illuminate early changes in health priorities and inform the design of timely health promotion strategies. Ultimately, these efforts can contribute to healthier populations and support the realization of SDG 3 in the post-pandemic world (Hales and Birdthistle, 2023).

## 2 Methods

### 2.1 Google Trends

Google Trends data was derived from a sample of Google search activities. The data were categorized, linked to specific topics, and anonymized. Searches containing special characteristics, those with insufficient search volumes, and repeated searches by the same individual within a short timeframe were excluded. Each sampled data point was scaled relative to the total number of searches within a specified location and period. This measure of “relative popularity” is expressed as the relative search volume (RSV), represented by a value between 0 and 100 (Google, 2009). RSV is displayed on a search volume index graph. Data were downloaded in comma separated values (CSV) file from Google Trends' website (https://trends.google.com/trends/) and are available on a weekly basis. Although Google Trends captures population-level information-seeking patterns rather than observable contact networks or transmission dynamics, its ability to reflect diffusion-like behavioral signals aligns conceptually with broader diffusion-modeling frameworks, including SEIR-based inference approaches (Sefer, 2022).

### 2.2 Documentation of Google Trends use

**Figure 1:**
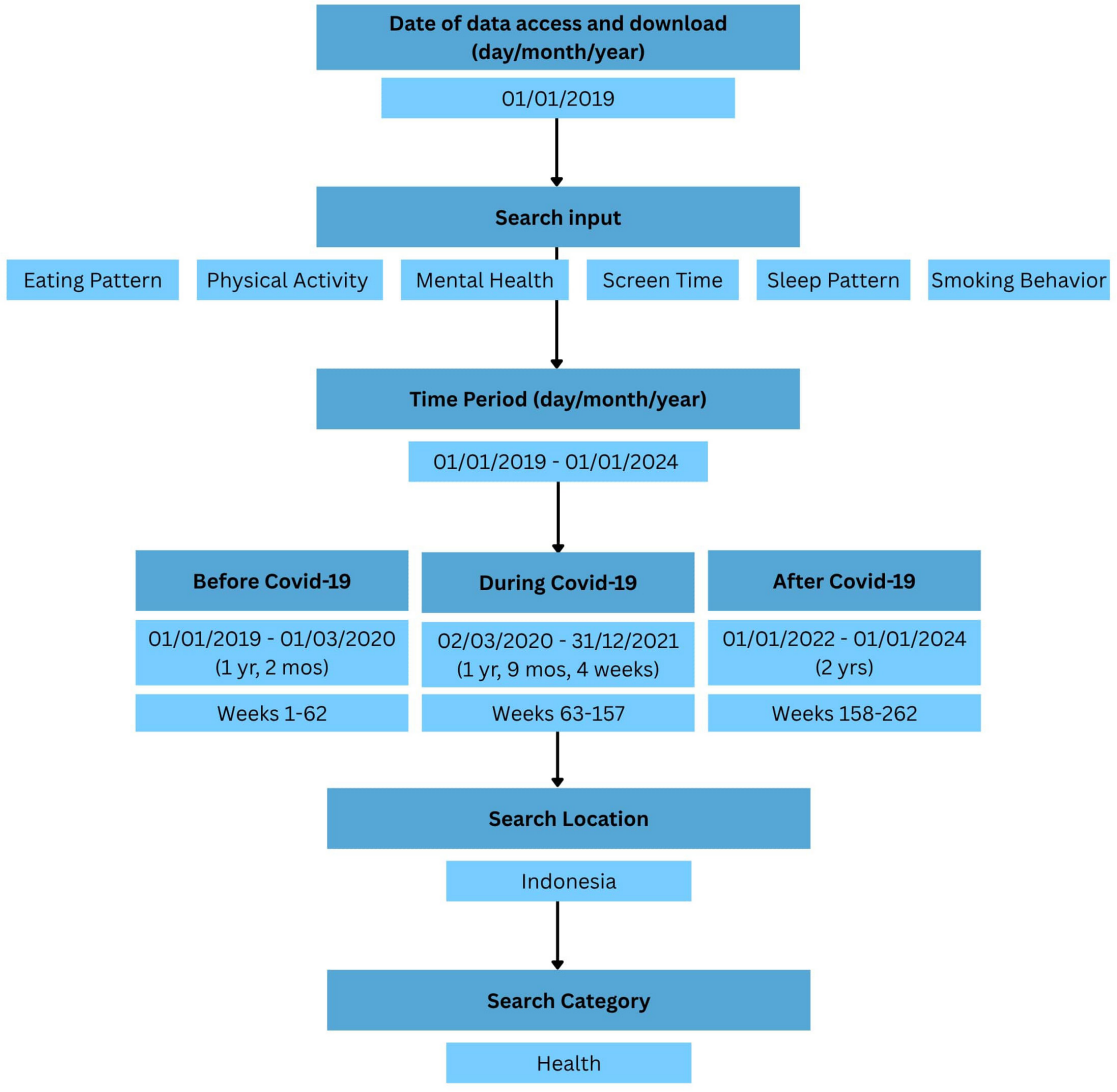
Visual schema of Google Trends.

Users can customize the different features of Google Trends to refine their searches. To maintain transparency, reproducibility, and methodological rigor, we adhered to the reporting guidelines proposed by Nuti et al. (2014). Figure 1 shows the schematic of the search strategy.

Google Trends data for this study were retrieved for January 2019–December 2023 to capture long-term patterns of public awareness across the pre-pandemic, pandemic, and post-pandemic phases in Indonesia. This multi-year window aligns with the epidemiological context in Indonesia, where the first confirmed COVID-19 cases emerged in early 2020, mobility restrictions were progressively relaxed between mid-2021 and early 2022, and population activities largely returned to baseline levels throughout 2022–2023 (Ariawan and Jusril, 2020; Singh et al., 2022).

Google Trends does not provide stable daily data for extended time spans; for multi-year periods, the platform automatically aggregates search activity into weekly Relative Search Volume (RSV) Weekly RSV represents normalized search interest rather than absolute query counts, following Google's internal sampling and re-normalization procedures. Prior methodological work has noted that these processes limit the feasibility of obtaining day-level stability for multi-year analyses, making weekly data the only methodologically reliable option for long-term infodemiological studies (Effenberger et al., 2020; Roudebush et al., 2025).

To address the inherent volatility of RSV time series, weekly data were analyzed using Joinpoint Regression, which identifies statistically significant changes in underlying trend segments and reduces the influence of short-term fluctuations. This technique identifies statistically significant changes in trend segments by modeling smoothed underlying patterns rather than relying on single-week fluctuations. Such smoothing-based approaches are frequently recommended when evaluating long-term digital epidemiology datasets. Given that the aim of this study was to examine broad, long-term awareness patterns rather than monitor real-time outbreak signals, the use of weekly multi-year RSV provides an appropriate and valid representation of population-level information-seeking behavior.

### 2.3 Search variables

Data will be accessed and downloaded from Google Trends, covering the period 2019 to 2023. The location of the search is Indonesia, aiming to capture broader trends in public awareness of “the big six” health behaviors. The timescale for each Google Trends query was divided into three distinct periods: pre-COVID-19 (January 2019 to February 2020), COVID-19 (March 2020 to December 2021), and post-COVID-19 (January 2022 to December 2023). This timeline will allow for the measurement of changes in public awareness across different phases of the pandemic.

To determine the behavioral domains analyzed in this study, we began with Life's Essential 8 (LE8), which includes four lifestyle components (diet, physical activity, nicotine exposure, and sleep) and four clinical components (Lloyd-Jones et al., 2022). As clinical factors cannot be meaningfully assessed through search-based infodemiologic data, the conceptual basis of this study focuses solely on lifestyle components. Building on this foundation, the study incorporated the modern “big six” lifestyle framework, which is widely applied in population health research, including WHO youth lifestyle guidance and Australian behavioral clustering studies (Gardner et al., 2020). The big six comprises diet, physical activity, smoking, sleep, screen time, and stress, thereby extending the lifestyle constructs of LE8 and reflecting contemporary behavioral risk patterns.

Integrating LE8 with the big six ensured that the selected domains remained grounded in an established cardiovascular health framework while also aligning with lifestyle issues that are highly relevant in Indonesia. The big six complements the lifestyle components of LE8 and captures behavioral concerns such as screen time and stress that increasingly shape public health discourse and generate substantial search activity. This combined framework provides a conceptually coherent and contextually appropriate basis for Google Trends–based analysis.

To assess public awareness of healthy lifestyle behaviors using Google Trends, data were extracted using the “topic” feature rather than conventional search terms. Search terms capture only the exact keywords typed by users, whereas topics aggregate semantically related queries across spelling variations, languages, and phrasings. In the preliminary stage, we tested several search terms for each behavioral domain, but many produced inconsistent or low RSV patterns, suggesting limited representativeness within Indonesia's multilingual context.

Using topics provided broader semantic coverage and stronger conceptual validity. For example, the search term “smoking” only captures queries containing that exact English word, whereas the topic “smoking” encompasses a wider set of semantically related queries across multiple languages, including Indonesian terms such as “*merokok,” “bahaya merokok,”* or “*rokok dan kesehatan*,” as well as English variants like “tobacco use” or “effects of smoking.” Given this ability to reduce semantic ambiguity and enhance data robustness, the topic-based approach was deemed more appropriate for the present infodemiologic analysis.

Within each health behavior domain, several related topic terms were initially tested as potential topic candidates. These were systematically compared in terms of and thematic content of their related queries, both of which reflect real-time user engagement and curiosity. The final selection of the topic terms for each domain was guided by three main criteria.

1. The clarity and representativeness of the topic in capturing the intended health behavior;
2. The consistency and prominence of RSV over time in the Indonesian context;
3. The relevance and richness of “related queries”, which provided qualitative insights into public concerns, practical questions, and behavioral intentions associated with each topic.

This method ensured that the selected topics not only reflected popular terminology, but also aligned with authentic public interests regarding health behavior during the post-pandemic recovery period. The full list of the tested topic terms and final topic selections per behavioral domain is presented in Table 1.

**Table 1:** Screening and final topic terms selection across behavioral domains.

**Behavioral domains**	**Topic terms input**	**Topic terms selection**
Dietary behavior	Health food, healthy diet, nutrition, food, and cuisine	Health food, nutrition, food
Physical activity	Exercise, physical activity, jogging, warming up, sports	Exercise, physical activity
Mental health	Anxiety (disorder), mental health, mental breakdown, depression, tension headache	Mental health, anxiety, depression
Screen time	Screen time, sedentary lifestyle, gadget, social media, dry eyes	Screen time, sedentary lifestyle, gadget
Sleep patterns	Insomnia, sleep, day time, nap, slow-wave sleep	Insomnia, sleep
Smoking behavior	Tobacco smoking, smoking, passive smoking, cigarettes, electronic cigarettes	Smoking, tobacco smoking

### 2.4 Selected topics

As summarized in Table 1, an initial pool of candidate Google Trends topics was generated for each of the six behavioral domains, and then refined to a final set that most faithfully represented public search behavior in Indonesia. Each behavioral category is discussed below, outlining the reasoning behind the inclusion of selected topics and exclusion of others based on both quantitative patterns and qualitative insights derived from related search queries.

The process of selecting the final topics revealed how the Indonesian public engaged in different aspects of health-related behavior during and after the pandemic. In the case of dietary behavior, the term “healthy diet” initially appeared promising owing to its high RSV. However, most of its related queries focused on weight loss products, slimming programs, and branded diet plans, indicating commercial interest rather than an awareness of healthy eating habits. In contrast, “health food” and “nutrition” were more aligned with everyday concerns, such as choosing balanced meals, understanding food quality, and meeting nutritional needs. The topic “food” although broad, turned out to be relevant because people searched for things like “what is healthy food?” and “how to eat well,” showing practical curiosity about healthy eating. Whilst, the term “cuisine” was excluded because most queries were about recipes, types of dishes, cooking styles, and non-health behaviors.

For physical activity, the topic “exercise” had the highest RSV and was clearly linked to behavior-oriented queries such as “how to start working out” or “exercise for fitness.” This shows strong public interest in taking action to improve physical health. The topic term “physical activity” was also chosen because the questions were still related to increasing movement and remaining active. Other topics, such as “jogging” and “warming up” had much lower RSVs and mostly showed specific or technical searches. The term “sports” were excluded because people searching for this term mostly looked for information about competition, teams, or athletes (topics outside the scope of daily lifestyle practices).

In the area of mental health, the selected topics (mental health, anxiety, and depression) reflected both general awareness and specific emotional concerns. While “mental health” covered broader searches about well-being and mental conditions, “anxiety” and “depression” brought in more personal and urgent queries such as “how to deal with anxiety” or “am I depressed?”. This indicates public struggles and the desire to seek help. Terms such as “mental breakdown” and “tension headache” had much lower RSVs and were often linked to more technical or medical discussions, rather than direct experiences or coping efforts.

For screen-related behaviors, a high RSV was found for “social media”, but this topic was excluded because most queries focused on influencers, content creators, or health advice on social media, not on screen use itself. Instead, topics such as “screen time” and “gadget” were more suitable because they captured concerns about overuse and the effects of prolonged digital exposure. The term “sedentary lifestyle” was also included because it was related to inactivity, and most of the queries talk about it caused by extended screen use. On the other hand, the topic “dry eyes” were excluded, not because of lack of relevance but because their related queries were vague and often unrelated to screen behavior, possibly tied to other medical conditions.

Insomnia and sleep were found to be the most relevant sleep patterns. The topic term “insomnia” was directly tied to behavioral struggles, with people searching for solutions like “how to sleep better” or “natural ways to treat insomnia”. The term “sleep” had the highest RSV and included general queries about sleep hygiene and irregular sleep routines, which are both important aspects of sleep behavior. Other terms, such as “nap”, “daytime”, and “slow-wave sleep”, had low RSV and were mostly technical or niche, not reflecting the everyday challenges that people faced.

Finally, for smoking behavior, the topics “smoking” and “tobacco smoking” were selected because of their high RSV and relevant search content. People are looking for information about smoking risks, how to quit smoking, and their long-term effects. The term “passive smoking” was associated with low search interest and mostly returned policy-related or conceptual results. “cigarettes” and “electronic cigarettes” were excluded because their queries focused on products, brands, or buying behavior and not on health awareness or behavior change.

### 2.5 Analytic methods

A time-trend analysis was conducted to examine changes in RSV as an indicator of online health information-seeking behavior (OHISB) related to the “big six” health behavior domains. Data were extracted in .csv format from the Google Trends Explore interface, separated by topic terms and restricted to Indonesia as the geographic filter, covering the period from January 2019 to December 2023.

The primary statistical method employed was joinpoint regression analysis, conducted using the Joinpoint Regression Program (version 5.3) developed by the U.S. National Cancer Institute. This technique identifies time points (joinpoints) at which statistically significant changes in linear trends occur. A maximum of three joinpoints was allowed for each model to align with the three pandemic phases while maintaining model interpretability. The optimal number of joinpoints was selected using a parametric permutation test comparing models with 0–3 joinpoints. Statistical significance was set at an alpha level of 0.05, and no logarithmic transformation was applied to the outcome variables.

Separate joinpoint regression models were fitted for each topic term. Slope estimates generated from each model quantified the absolute weekly change in RSV (0–100 scale) within each trend segment. These slopes represent the average increase or decrease in RSV per week during the specified interval. The statistical significance of each slope was evaluated to identify periods of meaningful trend shifts, with particular attention to whether a segment fell within the pre-, during-, or post–COVID-19 phases.

To provide mathematical transparency, each joinpoint model was formulated as a segmented linear regression of the form:


$$\begin{array}{l} RSV_{t}=\alpha +\beta t+\epsilon_{t}, \end{array}$$

where *RSV*_*t*_ represents the weekly Relative Search Volume at week *t*; β denotes the absolute weekly change in RSV; and ϵ_*t*_ is the error term. Because no logarithmic transformation was applied, the parameter β directly reflects the average absolute increase or decrease in RSV per week within each segment. Statistical significance for each slope was evaluated using a *t*-test:


$$\begin{array}{l} t=\frac{\beta }{SE\left(\beta \right)}. \end{array}$$

Model selection and joinpoint identification relied on the Monte Carlo permutation test implemented in the NCI Joinpoint Regression Program.

In addition to the joinpoint analysis, mean RSV values were compared across the three pandemic phases for each topic. This complementary analysis aimed to detect sustained shifts in public attention that may not manifest as abrupt trend changes. For each topic term, mean RSV differences (ΔRSV) across the three phases were calculated, and the Mann–Whitney U test was applied to assess the statistical significance of these differences.

## 3 Results

A joinpoint regression analysis was conducted to explore temporal changes in RSV across 15 topic terms representing the six health behavior domains. The analysis revealed that most topic terms experienced at least one statistically significant change in slope between 2019 and 2023. Table 2 summarizes the significant segments, direction of change, slope estimates, and *p*-values for each topic.

**Table 2:** Summary of significant slope changes by topic terms.

**Behavioral domain**	**Topics**	**Time segment with significant trend**	**Direction of Trend**	**Slope estimate (RSV/Week)**	***p***
Dietary behavior	Health food	Weeks 26–31	Increased	11.09	0.02
		Weeks 31–262	Decreased	−0.09	< 0.001
	Food	Weeks 1–16	Decreased	−0.96	0.006
		Weeks 16–31	Increased	0.96	0.019
		Weeks 35–262	Decreased	−0.05	< 0.001
	Nutrition	-	-	-	-
Physical activity	Exercise	Weeks 1–87	Increased	0.16	< 0.001
		Weeks 87–101	Increased	3.17	< 0.001
	Physical activity	-	-	-	-
Mental health	Anxiety	Weeks 94–172	Increased	0.35	0.003
		Weeks 175–262	Increased	0.07	< 0.001
	Mental health	Weeks 1–174	Increased	0.14	< 0.001
		Weeks 174–262	Decreased	−0.06	0.029
	Depression	Week 46–262	Decreased	−0.07	< 0.001
Screen time	Screen time	Week 1–262	Increased	0.18	< 0.001
	Sedentary lifestyle	Weeks 1–124	Increased	0.10	0.002
		Weeks 127–209	Increased	0.40	< 0.001
		Weeks 209–262	Decreased	−0.96	< 0.001
	Gadget	Weeks 42–49	Decreased	−7.05	< 0.001
		Weeks 49–262	Decreased	−0.02	< 0.001
Sleep patterns	Insomnia	Weeks 63–71	Increased	5.27	0.0016
		Weeks 71–81	Decreased	−3.45	0.0018
		Weeks 81–262	Decreased	−0.06	< 0.001
	Sleep	Weeks 64–72	Increased	3.20	0.006
		Weeks 72–82	Decreased	−1.94	0.013
		Weeks 82–262	Increased	0.06	< 0.001
Smoking behavior	Smoking	Weeks 71–214	Increased	0.21	< 0.001
		Weeks 214–262	Decreased	−0.46	< 0.001
	Tobacco smoking	Weeks 1–8	Increased	6.94	< 0.001
		Weeks 8–75	Decreased	−1.20	< 0.001
		Weeks 75–216	Increased	0.06	< 0.001
		Weeks 216–262	Decreased	−0.27	0.005

**Figure 2:**
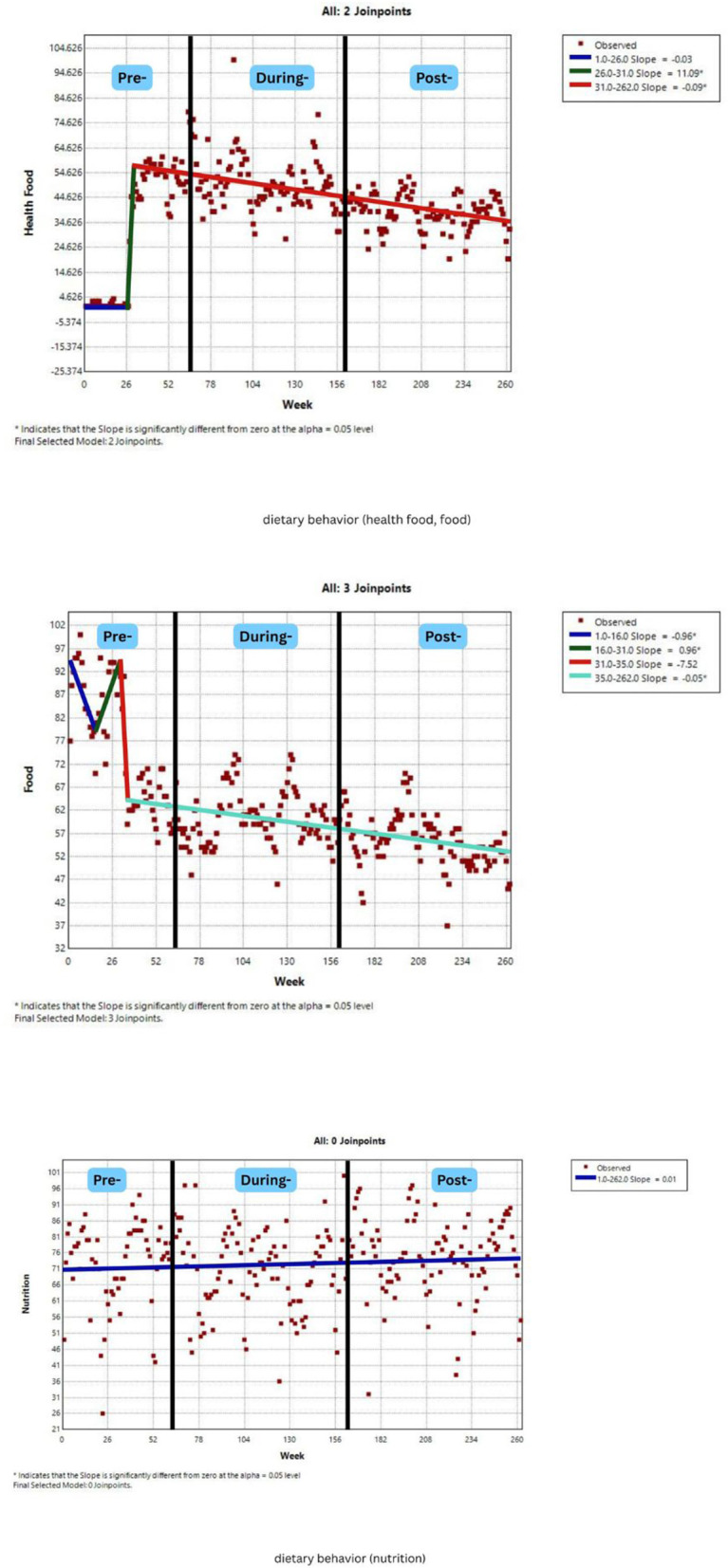
RSV trends for dietary behavior awareness over time. Slopes (blue, green, red, and mint green) represent trend segments identified by joinpoints. Black lines indicate the pre-, during-, and post-COVID phases.

**Figure 3:**
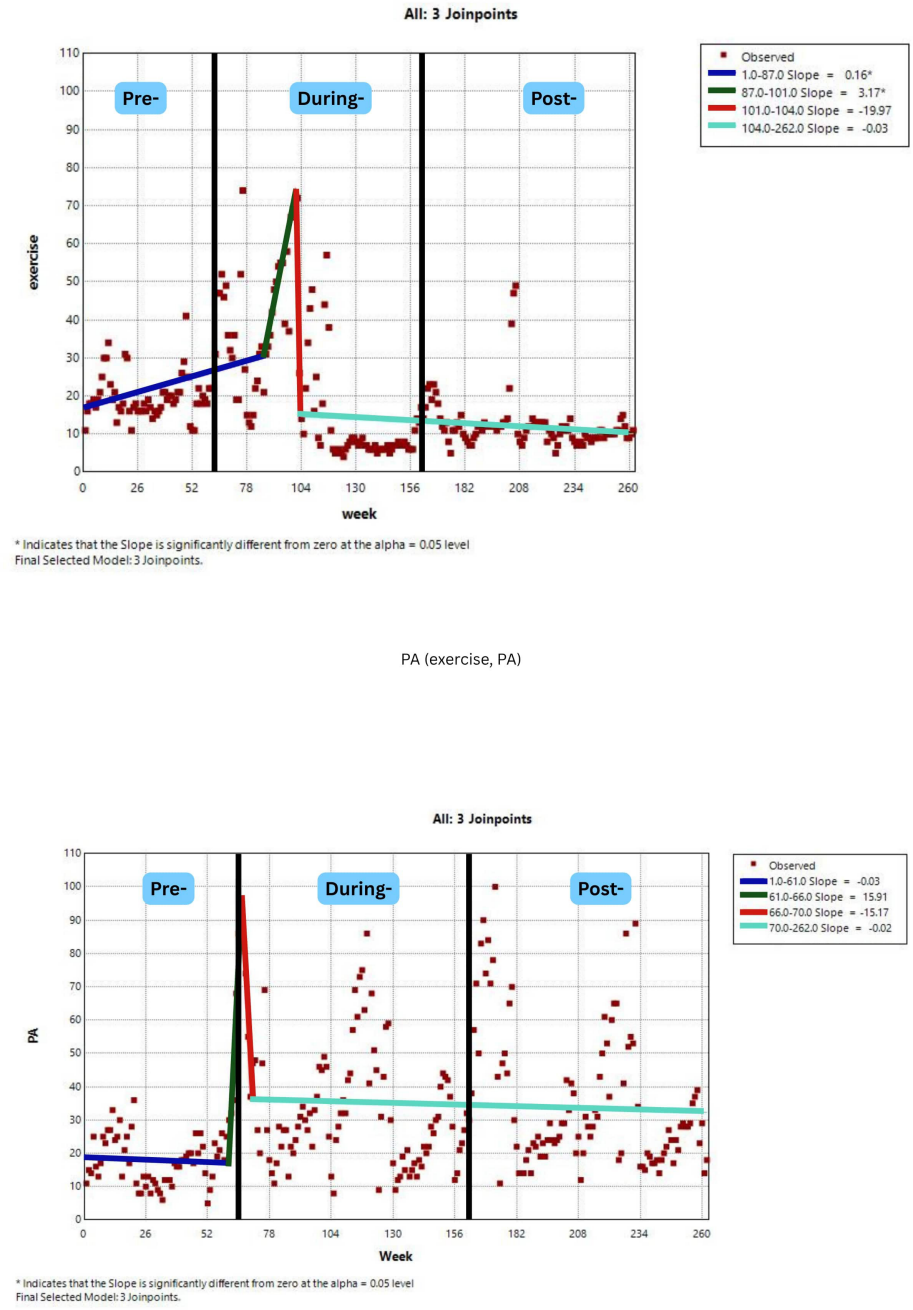
RSV trends for physical activity awareness over time. Slopes (blue, green, red, and mint green) represent trend segments identified by joinpoints. Black lines indicate the pre-, during-, and post-COVID phases.

**Figure 4:**
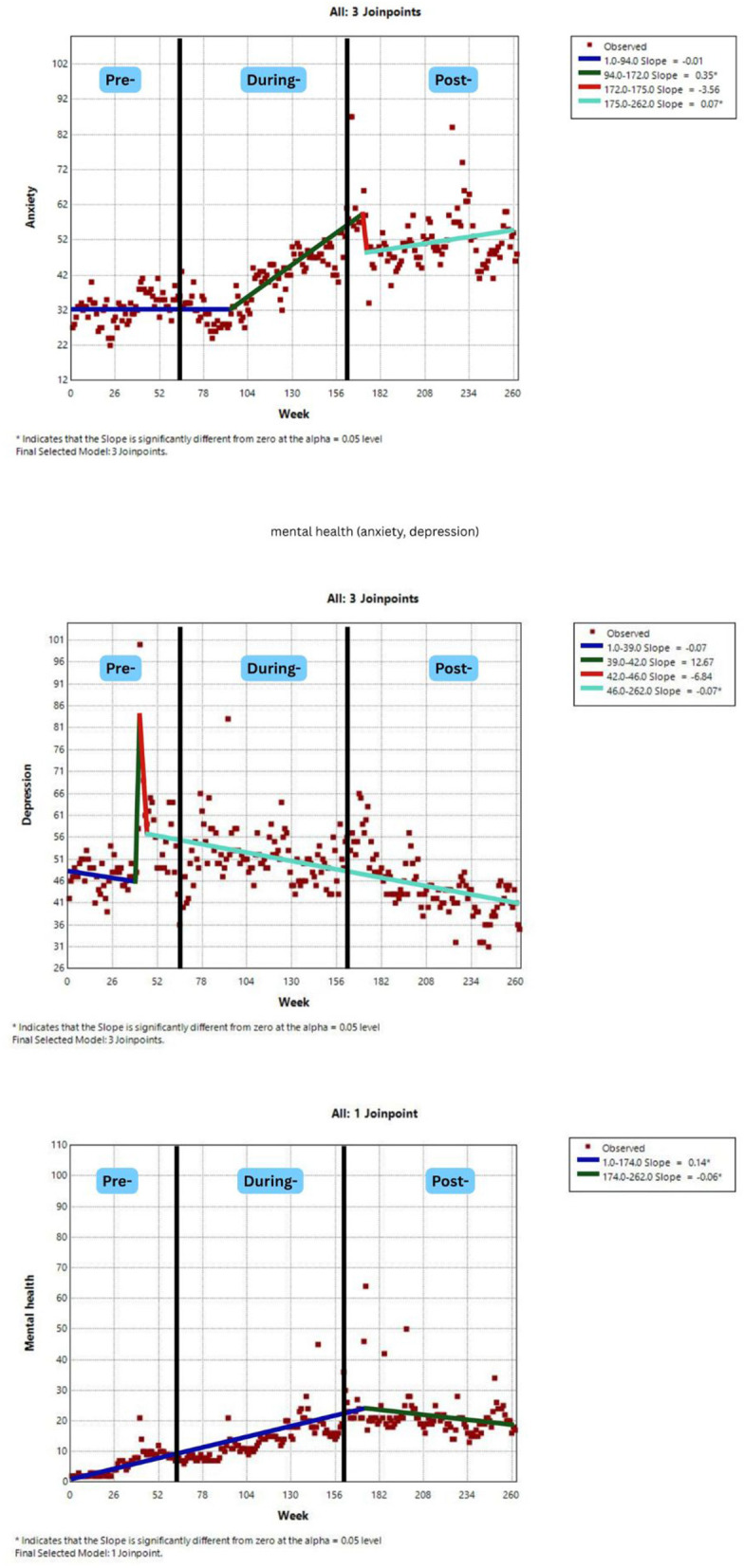
RSV trends for mental health awareness over time. Slopes (blue, green, red, and mint green) represent trend segments identified by joinpoints. Black lines indicate the pre-, during-, and post-COVID phases.

**Figure 5:**
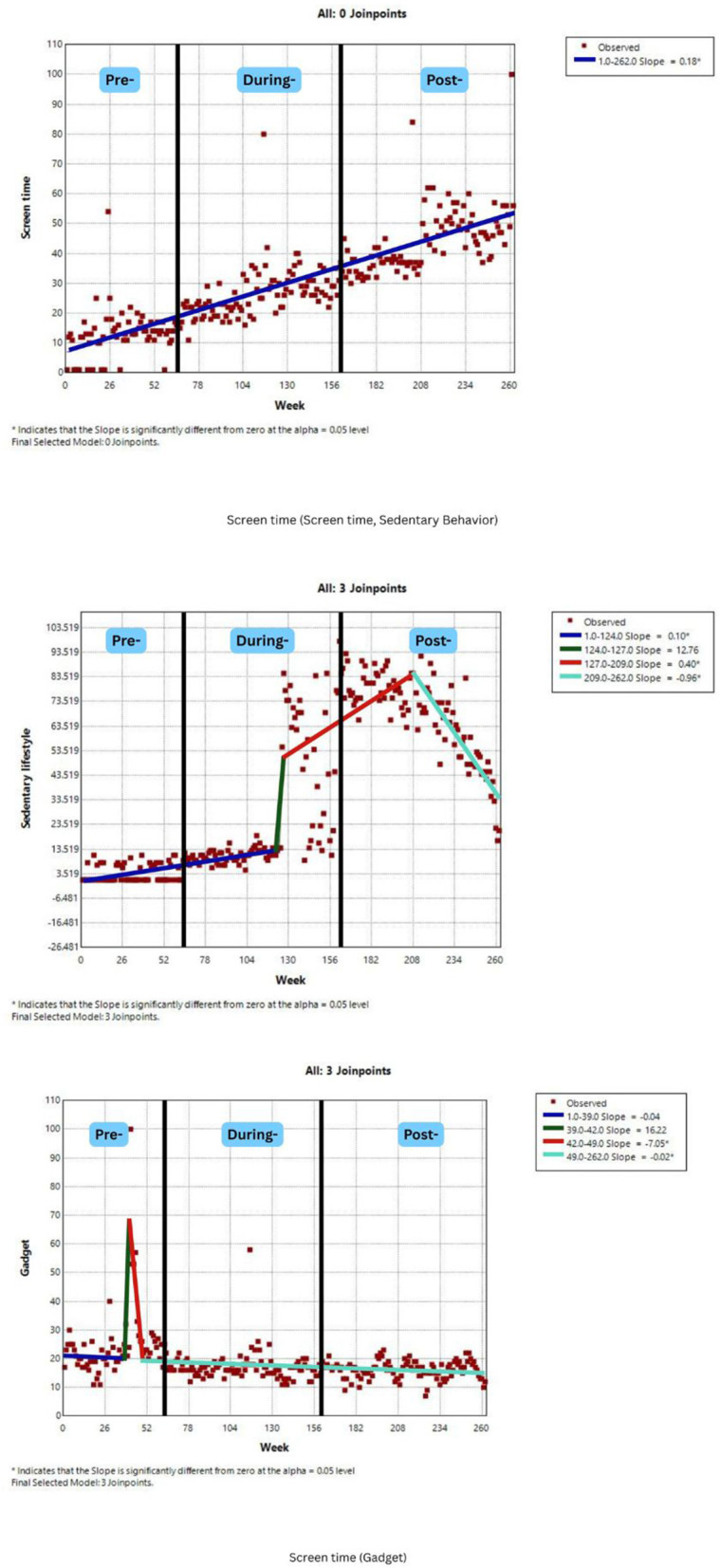
RSV trends for screen time awareness over time. Slopes (blue, green, red, and mint green) represent trend segments identified by joinpoints. Black lines indicate the pre-, during-, and post-COVID phases.

**Figure 6:**
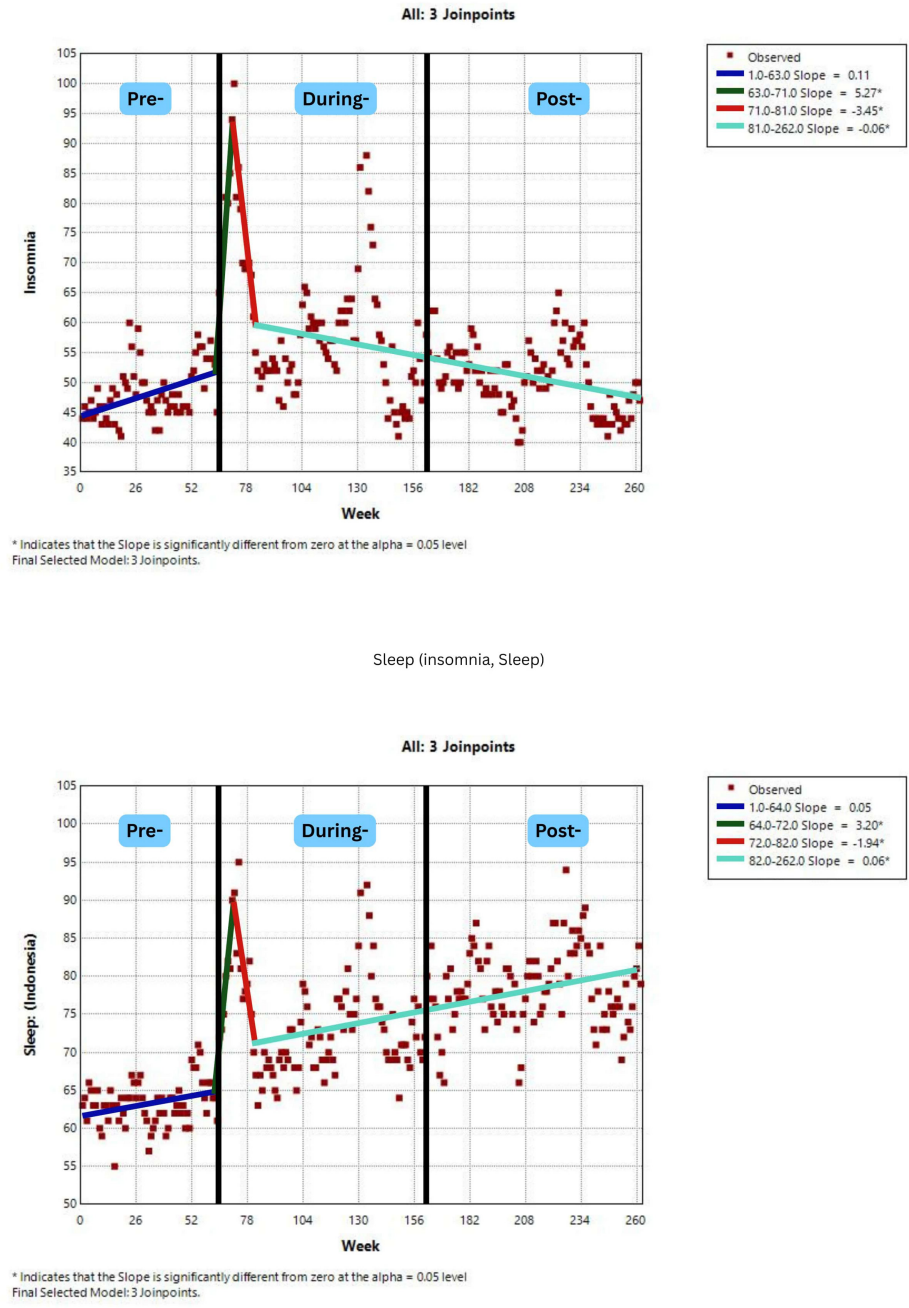
RSV trends for sleep patterns awareness over time. Slopes (blue, green, red, and mint green) represent trend segments identified by joinpoints. Black lines indicate the pre-, during-, and post-COVID phases.

**Figure 7:**
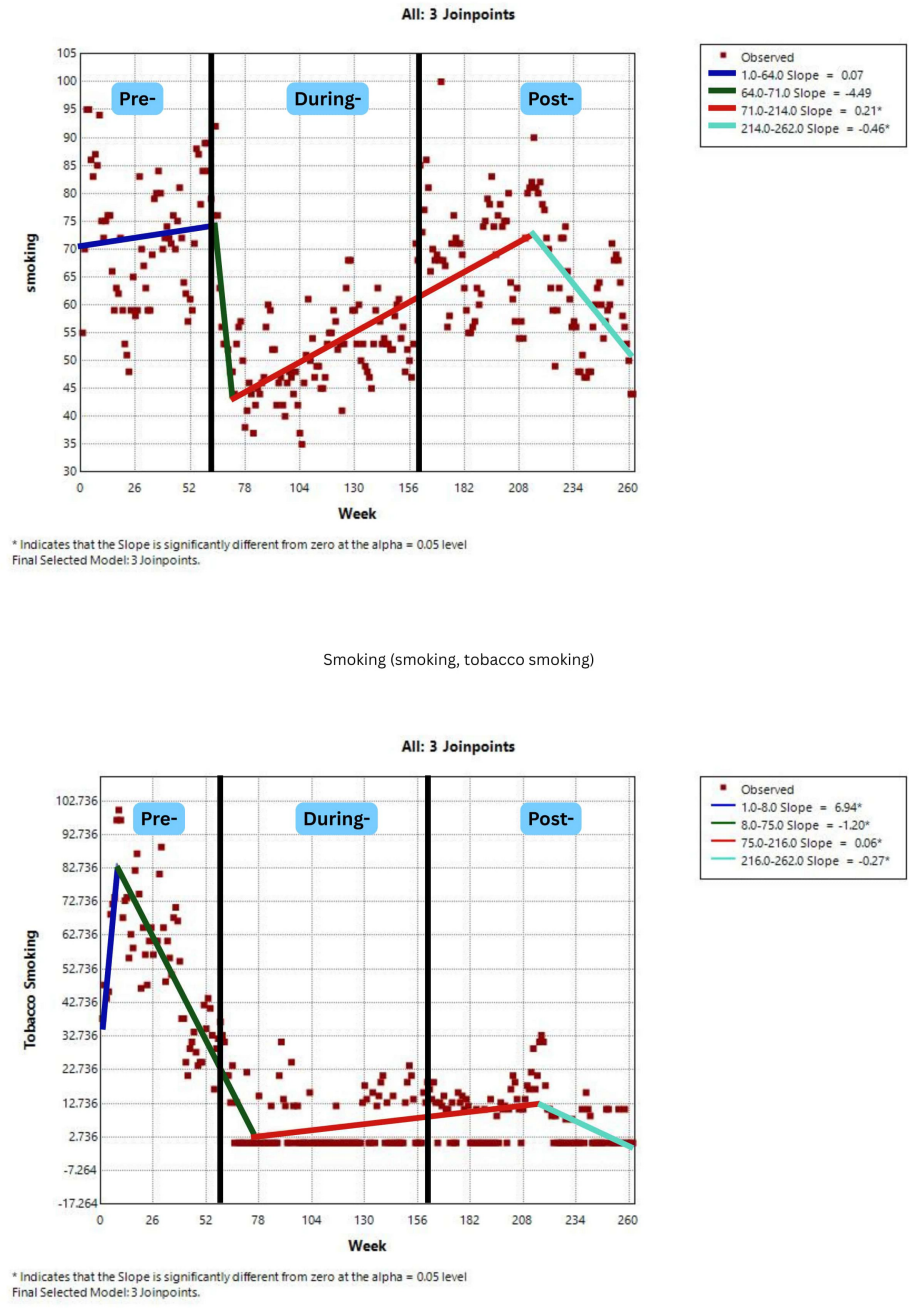
RSV trends for smoking behavior awareness over time. Slopes (blue, green, red, and mint green) represent trend segments identified by joinpoints. Black lines indicate the pre-, during-, and post-COVID phases.

Summary of significant trends based on joinpoint regression, corresponding to Figures 2–7. All slopes listed are statistically significant (*p* < 0.05) and represent weekly changes in RSV.

Figures 2–7 display individual joinpoint trend graphs for each topic term grouped by the behavioral domain. Comparisons were integrated within each behavioral section to provide clearer insight and avoid redundancy.

### 3.1 Dietary behavior

Three topic terms (health food, food, and nutrition) were analyzed for dietary behavior (Figure 2). Among them, the term “nutrition” showed no significant joinpoints, suggesting stable public attention over time. The topic term “health food” displayed two significant trend changes with an increase during weeks 26–31 (pre-COVID-19) and a sharp positive slope (11.09; *p* = 0.02), followed by a long-term decrease throughout weeks 31–262 (spanning late pre-, entire during-, and post-COVID-19 phases), with a small negative slope (−0.09; *p* < 0.001). Whilst, the term “food” had three joinpoints, a decrease in weeks 1–16 (pre-COVID-19), a short-lived increase in weeks 16–31 (still pre-COVID-19), and a consistent decline from week 35 to 262 (covering late pre- and post-pandemic). Overall, dietary behavior showed multiple joinpoints but predominantly declining trends, particularly after weeks 31 (pre-COVID-19 phases). This suggests that the public interest in dietary topics may have been short-lived and unsustained during or after the pandemic.

### 3.2 Physical activity

For physical activity, only the term “exercise” had significant joinpoints. There were two consecutive increasing trends, a gradual rise from weeks 1 to 87 (mostly pre- and early during COVID-19) with a slope of 0.16 (*p* < 0.001), followed by a sharp rise from weeks 87 to 101 (during COVID-19) with a slope of 3.17 (*p* < 0.001). These back-to-back positive trends both the pre-pandemic and during-pandemic periods indicate a growing interest in physical activity, particularly during the core COVID-19 period. Notably, there were no post-pandemic joinpoints, which may reflect a tapering off of new changes in attention after COVID-19. The presence of two strong positive joinpoints during COVID-19 highlights the impact of the pandemic on physical activity awareness (Figure 3).

Three mental health-related terms, namely “anxiety”, “mental health”, and “depression”, showed distinct trajectories (Figure 4). The topic term “anxiety” had two increasing trends, first from week 94 to 172 (during COVID-19) and then again from week 175 to 262 (post-COVID-19), indicating sustained and growing interest from mid-pandemic onward. In contrast, the term “mental health” increased steadily in weeks 1–174 (pre-and mostly during COVID-19) and then decreased in weeks 174–262 (transition from during to post-pandemic). The topic term “depression” showed only a long-term decrease from weeks 46 to 262 (covering late pre-, entire during-, and post-pandemic). Overall, mental health domains displayed the highest number of joinpoints with consistent upward trends for topic “anxiety”, emphasizing its salience during and after COVID-19. However, depression exhibited a downward trend, possibly due to a shift in public focus toward more specific emotional states such as anxiety.

### 3.3 Screen time

Public interest in term “screen time” showed a single long-term increase from across all three phases with a steady slope of 0.18 (*p* < 0.001), indicating constant growth without inflection points (Figure 5). In contrast, topic term “sedentary lifestyle” exhibited three trend changes with two increases covering pre-COVID-19 to the end of COVID-19 period and a decrease in weeks 209–262 (late post-COVID-19). Additionally, the term “gadget” often associated with digital addiction, showed two consistent declines, first sharply during COVID-19 and then more gradually to the rest of pandemic and post-pandemic. This domain showed mixed patterns, with screen time increasing steadily, sedentary lifestyle rising during COVID-19 and falling afterward, and gadget interest declining. The multiple joinpoints in the topic term “sedentary lifestyles” suggest that lifestyle-related awareness is more sensitive to pandemic dynamics than general screening behavior.

### 3.4 Sleep patterns

Public interest in topic “insomnia” demonstrated three distinct trend phases. There was a sharp increase in early COVID-19 period with a slope of 5.27 (*p* = 0.0016), followed by two successive declines at the end of pandemic to post-pandemic period, indicating a temporary concern that diminished over time. Whilst, the term “sleep” searches showed a similar initial surge at early pandemic, a decrease between weeks 72 to 82 (slope = −1.94, *p* = 0.013), and a long sustained increase in the middle of pandemic to the rest of post-pandemic period. These contrasting trajectories suggest that while acute sleep problems like insomnia declined after the pandemic peak, general public interest in sleep remained elevated, possibly reflecting a growing awareness of long-term sleep quality. The presence of multiple joinpoints in both topics highlights sleep as a behavior highly responsive to evolving stress and lifestyle patterns across the pandemic phases.

### 3.5 Smoking behavior

The topic term “smoking” showed two trend segments with an increase during pandemic to early post-pandemic period, followed by a decrease in weeks 214–262 (post-COVID-19). In contrast, the term “tobacco smoking” exhibited four shifts, a brief increase pre-COVID-19, a steep decrease in weeks 8–75 (pre to early COVID-19), a small increase during COVID-19, and a final decrease in post-COVID-19 period. This complex pattern, with frequent reversals, suggests that fluctuating attention is likely driven by factors beyond health concerns or possibly economic factors. This suggests that many people may still be unaware by the dangers of smoking (Figure 7).

### 3.6 Comparison of mean RSV across pandemic phases

To complement the segmented trend analysis, we examined changes in mean RSV across the pre-, during-, and post-pandemic phases. This analysis allowed us to capture broader shifts in public attention, particularly in cases in which joinpoint regression may not have identified abrupt slope changes. Table 3 presents the average RSV values and corresponding standard deviations for each topic term, along with the Mann–Whitney U test results comparing adjacent periods.

**Table 3:** Distribution of mean RSV for each time period.

**Behavior**	**Topic input**	**Mean RSV (SD)**			***p* (pre- and during)**	***p* (post- and during)**
		**Pre-COVID**	**During-COVID**	**Post-COVID**		
Dietary behavior	Health food	29.1 (25.4)	51.0 (10.9)	38.9 (6.8)	< 0.001^*^	< 0.001^*^
	Food	76.5 (13.0)	60.2 (5.8)	55.4 (5.5)	< 0.001^*^	< 0.001^*^
	Nutrition	72.1 (13.3)	70.2 (11.5)	74.5 (12.1)	0.215	0.012^*^
Physical activity	Exercise	19.6 (5.7)	24.2 (21.1)	12.4 (6.6)	0.436	0.133
	Physical activity	17.8 (7.2)	35.2 (17.4)	35.1 (20.6)	< 0.001^*^	0.816
Mental health	Anxiety	32.6 (4.2)	38.4 (7.9)	52.9 (9.7)	< 0.001^*^	< 0.001^*^
	Mental health	6.0 (3.8)	14.2 (11.2)	21.8 (7.0)	< 0.001^*^	< 0.001^*^
	Depression	51.7 (9.4)	51.0 (5.9)	45.2 (7.1)	0.354	< 0.001^*^
Screen time	Screen time	12.7 (8.1)	25.6 (8.5)	43.9 (10.9)	< 0.001^*^	< 0.001^*^
	Sedentary lifestyle	3.7 (3.6)	23.9 (17.4)	68.3 (17.4)	< 0.001^*^	< 0.001^*^
	Gadget	25.1 (13.0)	17.2 (5.6)	16.0 (3.0)	< 0.001^*^	0.279
Sleep	Insomnia	48.2 (4.6)	60.4 (11.2)	50.9 (5.6)	< 0.001^*^	< 0.001^*^
	Sleep	63.2 (2.9)	74.0 (7.2)	78.1 (5.2)	< 0.001^*^	< 0.001^*^
Smoking behavior	Smoking	72.0 (11.6)	52.1 (6.9)	65.8 (10.9)	< 0.001^*^	< 0.001^*^
	Tobacco smoking	53.2 (20.8)	6.32 (7.8)	8.54 (7.9)	< 0.001^*^	0.042^*^

Mann-Whitney U test was used to compare RSV values between two time periods.

^*^p < 0.05 indicates statistical significance.

Among the dietary-related terms, only the term “health food” demonstrated a significant mean difference rising from 29.1 in pre-COVID-19 to 51.0 during COVID-19 period. The narrower standard deviation (SD) during this period suggests that the increase was not only higher on average but also more evenly distributed across weeks, indicating a widespread and consistent rise in public awareness. Although RSV declined slightly post-pandemic, it remained well above baseline levels, suggesting residual interest. In contrast, both term “food” and “nutrition” did not follow the same trajectory. Food intake decreased significantly across all three phases, from 76.5 to 55.4, with progressively narrower SDs pointing to a uniform decline rather than sporadic peaks. Whilst, the term “nutrition” remained relatively stable across periods, showing only a modest but significant difference between the COVID-19 and post-COVID-19 phases, reflecting less responsiveness to pandemic-related shifts.

In the physical activity domain, both the term “exercise” and “physical activity” increased during the COVID-19 pandemic, but only the latter showed a significant difference. While the term “exercise” rose from 19.6 to 24.2 with a very high SD during COVID-19 suggests fluctuating interest, possibly tied to episodic trends. Interest dropped again after the pandemic (12.4), showing no sustained engagement. In contrast, the term “physical activity” increased markedly, from 17.8 to 35.2, and remained elevated afterward, indicating a consistent increase in awareness. However, large SDs in both periods indicate varied weekly engagement.

All topic terms in the mental health domain showed significant differences during the pandemic, although they had different trajectories. The topic term “anxiety” rose steadily across all three phases, with low SDs indicating consistently high and widespread public attention. Additionally, the term “mental health” also showed a significant increase, though the higher SD during COVID-19 reflects more variable weekly search behavior. In comparison, the topic term “depression,” did not differ significantly between pre- and during COVID-19 periods, but declined significantly post-pandemic. This suggests that while depression was already a concern before COVID-19, public attention may have shifted toward more acute mental health issues such as anxiety in the following years.

Searches related to screen-based behaviors also showed a statistically significant increase. The term “sedentary lifestyle” rose sharply from 3.7 to 68.3 post-pandemic, with stable SDs, indicating sustained and widespread public concern. Similarly, the topic term “screen time” increased steadily across phases (12.7–43.9), and moderate SDs suggest consistent growth in public attention. In contrast, the term “gadget” searches that often associated with digital addiction, declined over time with narrowing SDs from 13.0 to 3.0, implying that public interest in device-related content became more limited and less diverse.

Sleep-related terms also showed significant differences. The topic term “insomnia” rose during the pandemic 48.2–60.4 before declining slightly after COVID-19. Although SDs were higher during COVID-19, the trend points to a heightened and fluctuating awareness of sleep disturbances. In contrast, the term “sleep” showed a steady increase throughout the three phases, from 63.2 to 78.1%, with consistently low SDs, indicating a growing and stable interest in sleep quality over time.

Smoking-related searches have complex patterns. The topic term “smoking” declined significantly during COVID-19, then rose again post-pandemic, though not to baseline levels. Narrowing SDs indicates more uniform engagement in later periods. Whilst, topic term “tobacco smoking” dropped sharply during COVID-19 from 53.2 to 6.3 and showed only minimal recovery afterward (8.5); both means in each time period were significantly different. These findings suggest that while general terms such as smoking regained attention post-COVID-19, more clinical terminology such as tobacco smoking remained underutilized, highlighting potential limitations in the public's engagement with formal health messaging even in the context of a global health crisis.

## 4 Discussion

The COVID-19 pandemic offered a critical moment for public re-engagement with health behaviors, yet digital interest patterns revealed a fragmented and often inconsistent response. Across behavioral domains, our analysis shows that increases in awareness often did not translate into meaningful or sustained changes in real-world behavior. This disconnect suggests that while digital search data can offer early signals of public concern, awareness alone is rarely sufficient to catalyze long-term behavioral change, especially without supportive structural, cultural, or economic factors.

Dietary behavior exemplifies this mismatch between awareness and action. Although nutrition was widely promoted during the pandemic as a means to boost immunity, our results showed no upward joinpoints and only a limited increase in mean RSV. This stagnation was echoed in real-world data. Basic Health Survey reported that fruit and vegetable consumption remained critically low, declining from 4.6% to 3% (Ministry of Health Indonesia, 2018, 2023), and 96% of respondents continued prioritizing taste over health motivations. These figures suggest that dietary awareness did not penetrate deeply enough to alter behavior. Contributing factors likely include entrenched cultural norms and structural challenges such as affordability and access (Darmon and Drewnowski, 2015). While some individuals reported an uptick in home cooking or supplement use (Anyanwu et al., 2022; Riyanto et al., 2023), these appear to be transient responses rather than lasting transformations. The decline in nutrition-related search interest supports prior findings that crises often deprioritize healthy eating in favor of emotional comfort and accessibility (Monteiro et al., 2018; Muscogiuri et al., 2020). In comparison, physical activity initially benefitted from a surge in awareness, with a notable spike in the term “exercise” searches during early lockdown periods. This coincided with public messaging around the benefits of movement for immunity and stress management (Sitohang and Ghani, 2021). However, this digital engagement did not persist post-pandemic, and actual behavior fell short of this early momentum. National data indicate a slight increase in physical inactivity rates, and reports show that children became increasingly sedentary due to school closures and device overuse (Andriyani et al., 2021; Ministry of Health Indonesia, 2023). This illustrates how even when awareness exists, behavior is constrained by environmental and systemic limitations, confirming Robillard, Dion, Pennestri, Solomonova, Lee and Saad (2021) assertion that behavior change requires reinforcement beyond individual intent. In such cases, digital interest may serve as an indicator of momentary curiosity or intention, rather than a driver of sustained action (Robillard et al., 2021).

Mental health awareness told a different story. It was the only domain that experienced a robust and sustained increase in digital engagement throughout the pandemic. Search trends paralleled growing psychological distress, with studies reporting anxiety rates from 20% to over 60% in Indonesia (Anindyajati et al., 2021; Romadhona et al., 2021; Sunjaya et al., 2021). In comparison, self-reported depression in SKI 2023 was only 1.4%, pointing to a significant discrepancy. This divergence may stem from ongoing stigma, underdiagnosis, or normalization of mental distress (Wade et al., 2021). The internet became a key space for emotional expression and information-seeking during limited access to care, and increased search volume may reflect hidden distress rather than clinical diagnoses (Naslund et al., 2020). This pattern is consistent with evidence showing that Google Trends does not reliably mirror population-level changes in depression, anxiety, self-harm, or suicidal ideation during public health emergencies, with several mental health–related Topics exhibiting inverse or non-significant associations with validated clinical measures (PHQ-9, GAD-7) (Knipe et al., 2021).

In this case, digital awareness may actually provide a more accurate representation of public health concern than official reporting systems. Behaviors related to sedentary lifestyle and sleep further complicate the awareness-behavior relationship. While the term “sedentary lifestyle” searches rose sharply, topic “screen time” followed a more gradual, linear pattern. This suggests not a sudden concern, but rather an acceptance of screen-heavy routines as the new norm, possibly due to prolonged remote work and learning. Similarly, both topic term “insomnia” and “sleep” search trends increased during the pandemic, with the latter maintaining elevated levels even after COVID's peak (Bhat and Chokroverty, 2022; Robillard et al., 2021). This persistence may indicate a growing prioritization of sleep hygiene, supported by evidence linking sleep to immune function and mental health (Alfonsi et al., 2022). However, these behavioral intentions may still be difficult to act upon in the absence of structural supports or lifestyle changes. Thus, even in domains with growing digital concern, the pathway from awareness to action remains obstructed. Smoking behavior adds another layer to this complexity. Although tobacco-related search interest declined during the pandemic, closer analysis shows that economic hardship played a larger role than health motivation. Recent work using Google Trends also reported a broad decrease in search interest for smoking-related keywords across multiple countries during the pandemic, with significant declines observed for 58% of cessation and 58% of consumption queries (Jagomast et al., 2024). Up to 40% of smokers reportedly reduced consumption due to financial pressure, with 25% switching to cheaper brands (Bella et al., 2023). The national smoking prevalence barely declined, from 24% to 22.4% (Ministry of Health Indonesia, 2018, 2023), and search interest in technical terms such as “tobacco smoking” diminished. This suggests that lower tobacco use was driven more by necessity than by health literacy (Kalkhoran et al., 2022; Klemperer et al., 2020), and without sustained cessation support, this pattern may be temporary.

Taken together, these findings reaffirm that digital awareness is not a reliable proxy for behavior. However, it would be a mistake to dismiss digital trends as irrelevant. In fact, spikes in search volume often reflect heightened concern triggered by personal, familial, or community-level experiences. In this sense, digital interest may serve as a powerful “early signal” for health promoters, offering clues about the lived experiences and latent anxieties of the population. For example, a sudden surge in topic term “insomnia” searches may precede formal reporting of stress-related conditions, offering a window of opportunity for timely intervention. The divergence between short-term curiosity and sustained attention becomes clearer when the slope patterns are interpreted alongside these behavioral signals. Rapid spikes that quickly reversed such as the brief surge in health food searches or the short-lived rise in sedentary lifestyle reflect situational, externally driven attention that fades once immediate pressures subside. In contrast, the persistent growth in anxiety-related searches and the stabilization of sleep-related interest point to deeper, emotionally anchored concerns that continue beyond the pandemic period. Here, the rate of decay in RSV acts as a meaningful indicator: steep declines represent event-driven curiosity, whereas slow or absent decay suggests durable health needs that resonate with the population. This distinction illustrates why some health topics experience only fleeting motivation, while others persist due to personal relevance, aligning with the well-documented intention–behavior gap.

For health promotion practice, this highlights an important implication: we must listen more closely to what people are searching. Digital behavior is not just noise, it is often a reflection of unmet needs, unspoken fears, and emerging problems. By integrating digital surveillance with behavioral insights and contextual data, we can design more responsive and proactive health campaigns. As shown in this study, digital awareness can help flag when and where deeper structural interventions are urgently needed. This study has several limitations. Google Trends restricts weekly-level data to a maximum of 5 years, resulting in an uneven distribution across pre-, during-, and post-pandemic periods. While using “topics” instead of specific search terms allows broader query aggregation, topic selection is limited to Google's internal suggestions, which may exclude relevant but less visible terms. Additionally, joinpoint analysis could not include standard errors or confidence intervals, as Google Trends does not provide raw data or variance estimates. Finally, the findings were not validated against real-world behavioral data, limiting inferences about actual behavior change.

## 5 Conclusion

This study found that digital interest in health-related behaviors varied considerably across domains during and after the COVID-19 pandemic. Mental health and sleep were the only topics that showed sustained increases in both weekly trends and mean RSV, suggesting a lasting shift in public concern. In contrast, awareness of physical activity rose sharply during the pandemic but declined afterward, while interest in dietary behavior and tobacco use showed no meaningful improvement. These findings suggest that only certain health concerns, especially those directly experienced during the crisis, triggered long-term public engagement. Leveraging digital surveillance tools like Google Trends can help identify which areas of health promotion gain traction over time, allowing future interventions to focus on domains where awareness is growing and sustained.

## Data Availability

The raw data supporting the conclusions of this article will be made available by the authors, without undue reservation.
